# Editorial: From Development to Senescence, Bridging the Gap in Lung Fibrosis

**DOI:** 10.3389/fmed.2021.798164

**Published:** 2021-11-18

**Authors:** Jason J. Gokey

**Affiliations:** Vanderbilt University Medical Center, Nashville, TN, United States

**Keywords:** interstitial lung disease (ILD), idiopathic pulmonary fibrosis, lung fibrosis, senescence, developmental pathways

This Research Topic titled “From Development to Senescence, Bridging the GAP in Pulmonary Fibrosis” sought to clearly define the role of developmental and senescence pathways that are abnormally regulated during the progression of interstitial lung diseases (ILDs). In contrast to the progress made in reducing the mortality in several other diseases including cancers, ILD mortality rates have remained constant over the last decade. ILDs are characterized by the loss of normal lung regions that are replaced by fibrotic tissue that replace normal alveoli leading to progressive respiratory failure and potentially death. The term ILDs is associated with several lung diseases including cystic fibrosis, scleroderma, sarcoidosis, and one of the most common forms of ILD, Idiopathic Pulmonary Fibrosis (IPF) (1). IPF is a rapidly progressing fibrotic disease in which patients, usually diagnosed in their 60s, have a poor prognosis, as the disease is usually fatal within 5 years of diagnosis (2, 3). The underlying mechanisms of IPF remain unclear, however repetitive injury and failure of alveolar repair leading to fibrotic activation have been associated with disease progression, and several single cell sequencing studies have identified aberrantly regulated pathways in the fibrotic lung. Nemeth et al. have sought to collect and collate the current knowledge generated on IPF disease development and progression through a review on the cellular heterogeneity during the progression and development of fibrotic lung disease and how these single cell studies provide insight toward identifying novel therapeutics for the treatment of IPF.

Several pathways have been implicated in driving development and progression of pulmonary fibrosis. Single cell sequencing has identified that the Hippo/YAP pathway is aberrantly activated in IPF. The recently identified role for YAP during normal alveolar development and repair, as well as aberrant activation leading to abnormal repair that induces atypical cell phenotypes associated with pulmonary fibrosis. Open questions surrounding the function of Hippo/YAP signaling in both normal and pathologic alveolar repair and fibroblast activation have been summarized by Gokey et al. One of the major pathways identified through genetic studies in mice has been the transforming growth factor-beta (TGFb) pathway. Al-Mudares et al. have sought to clarify the potential role of a TGFb pathway member growth differentiation factor 15 (GDF15), a protein that is ubiquitously expressed, that appears to play a role across the lifespan, as it has been implicated in early life diseases such as bronchopulmonary dysplasia to diseases associated with increased age such as COPD and IPF. The potential role of GDF15 in senescence points to another established risk factor of pulmonary fibrosis, increased age. The link between aging, exposure to injury, and senescence is further explored as a driving factor in onset of pulmonary fibrosis, which is increasingly critical as the world's population ages. Venosa defines the loss of normal cellular “fitness” through the progression of cellular senescence and discusses how as the patient ages, a major indicator of pulmonary fibrosis prognosis, the threshold of injury required to generate an irreversible phenotype is lowered, thereby increasing the potential for abnormal lung regeneration and onset of fibrosis. The link to chronic injury, and potential to treat the source of certain injuries is further explored by Duckworth et al., who collated current data on the role of herpes viral injury in the development of IPF and seek to clarify discrepancies as to whether herpes virus injury is a contributing factor. The potential mechanisms by which herpes virus induces fibrotic progression and whether treatment of viral injury may be beneficial in delaying fibrotic progression are discussed. In the current global environment, the potential role of viral infections leading to onset of pulmonary fibrosis is of eminent concern. The role of lung injury driven by Severe Acute Respiratory Syndrome coronavirus 2 that causes COVID-19, that leads to fibrotic remodeling in some patients is further explored by Kiener et al. The authors explore the various potential models available to study lung fibrosis *in-vitro* in human samples including the use of precision cut lung slices and organoids to study alveolar regeneration.

Understanding the mechanisms regulating alveolar repair, and determining ways to enhance alveolar regeneration, are beneficial approaches to identify potential efficacious treatments to delay or resolve pulmonary fibrosis. The most utilized model of pulmonary fibrosis is the use of bleomycin injury in mammalian models. Lopez-Rodriguez et al. have generated a novel study by which enhanced repair is capable in the bleomycin induced lung injury model. Instillation of primary AT2 cells into the lung 7 days post injury enhanced lung physiology compared to animals that did not receive AT2 cells when assessed at 14 days post injury.

Collectively these manuscripts collate and clarify the current literature in the field that defines the role of developmental and senescence pathways in the onset and progression of pulmonary fibrosis. This collection of timely and insightful reviews greatly assists the field in quickly organizing the current literature and providing novel insights that facilitates the advancement of the understanding of pathways and tools that may provide efficacious targets for delaying, preventing, or resolving pulmonary fibrosis and provide hope for patients diagnosed with this disease and its bleak prognosis.

## Author Contributions

The author confirms being the sole contributor of this work and has approved it for publication.

## Funding

This work was made possible by the Pulmonary Fibrosis Foundation, by an independent grant from Boehringer Ingelheim Pharmaceuticals, Inc. who provided the financial support. The author meets criteria for authorship as recommended by the International Committee of Medical Journal Editors (ICMJE) and was fully responsible for all aspects of the trial and publication development.

## Conflict of Interest

The author declares that the research was conducted in the absence of any commercial or financial relationships that could be construed as a potential conflict of interest.

## Publisher's Note

All claims expressed in this article are solely those of the authors and do not necessarily represent those of their affiliated organizations, or those of the publisher, the editors and the reviewers. Any product that may be evaluated in this article, or claim that may be made by its manufacturer, is not guaranteed or endorsed by the publisher.
